# Presence and Effects of Pituitary Adenylate Cyclase Activating Polypeptide Under Physiological and Pathological Conditions in the Stomach

**DOI:** 10.3389/fendo.2018.00090

**Published:** 2018-03-19

**Authors:** Dora Reglodi, Anita Illes, Balazs Opper, Eszter Schafer, Andrea Tamas, Gabriella Horvath

**Affiliations:** ^1^Department of Anatomy, MTA-PTE PACAP Research Team, Centre for Neuroscience, University of Pecs Medical School, Pecs, Hungary; ^2^1st Department of Internal Medicine, University of Pecs Medical School, Pecs, Hungary; ^3^Department of Gastroenterology, Medical Centre, Hungarian Defence Forces, Budapest, Hungary

**Keywords:** PACAP, stomach, secretion, motility, cancer, ulcer, gastritis

## Abstract

Pituitary adenylate cyclase activating polypeptide (PACAP) is a multifunctional neuropeptide with widespread occurrence throughout the body including the gastrointestinal system. In the small and large intestine, effects of PACAP on cell proliferation, secretion, motility, gut immunology and blood flow, as well as its importance in bowel inflammatory reactions and cancer development have been shown and reviewed earlier. However, no current review is available on the actions of PACAP in the stomach in spite of numerous data published on the gastric presence and actions of the peptide. Therefore, the aim of the present review is to summarize currently available data on the distribution and effects of PACAP in the stomach. We review data on the localization of PACAP and its receptors in the stomach wall of various mammalian and non-mammalian species, we then give an overview on PACAP’s effects on secretion of gastric acid and various hormones. Effects on cell proliferation, differentiation, blood flow and gastric motility are also reviewed. Finally, we outline PACAP’s involvement and changes in various human pathological conditions.

## Introduction

PACAP was isolated as a neuroendocrine regulator of the hypothalamo-hypophyseal system in two forms, PACAP1–27 and PACAP1–38, with 27 and 38 amino acid residues, respectively. PACAP and VIP belong to the same peptide family (VIP-secretin-glucagon), based on the structural similarity between the shorter form of PACAP and VIP. Later PACAP was shown throughout the body with a diverse array of functions, including the gastrointestinal system. VIP has long been known as a gastrointestinal peptide, but shortly after the isolation of its related peptide many, partially overlapping, gastrointestinal effects of PACAP have also been described. PACAP, similarly to VIP, occurs along the entire digestive tract, and both peptides are abundantly expressed in the enteric nervous system (ENS) and enteroendocrine cells, influencing secretion, motility and reflexes. PACAP acts through its specific PAC1 receptor and the VPAC1 and 2 receptors, which also bind VIP with similar affinity (1).

In the small and large intestine, PACAP is involved in several biological processes. Its effects on cell proliferation, secretion, motility, gut immunology, and blood flow have been shown (1–3). Under pathological circumstances, it has been shown that PACAP decreases inflammatory reactions both in the small and in the large intestine, while lack of PACAP is associated with increased inflammatory reactions and colon cancer development (4–6). The intestinal effects of PACAP have been reviewed several times by different authors (7–10). However, no current review is available on the actions of PACAP in the stomach in spite of dozens of articles published on the gastric presence and actions of the peptide. Therefore, the aim of the present review is to summarize currently available data on the distribution and effects of PACAP in the stomach.

## Presence and Distribution of PACAP and Its Receptors in the Stomach

The presence and distribution of PACAP have been studied in several species with different methods, and all have found PACAP expression in the stomach wall, with some qualitative and quantitative differences between species and methods used (11). Soon after its discovery, a radioimmunoassay (RIA) study described the distribution of PACAP in various tissues of the rat and found that PACAP1–38 was the dominant type in mammalian tissues. They could detect PACAP also in the gastrointestinal tract, with stomach, duodenum and jejunum showing higher levels compared to other parts of the intestinal system (12). In another study in rats, Hannibal et al. found high levels of PACAP1–38 in the stomach by RIA and lower levels of PACAP1–27 and PACAP-related protein. There was no difference between levels measured in the esophagus and antrum/fundus parts of the stomach, but higher levels were measured in the small intestine (11). The dominance of PACAP1–38 was also confirmed in the antrum part of the porcine stomach by another RIA study, with PACAP1–27 just reaching detection limit (13). The authors measured mucosal and muscularis extracts separately and found slightly higher levels in the muscularis part.

The first comparative immunohistochemical description of PACAP1–27-like immunoreactivity in the alimentary canal of several species came from Sundler et al. (14), who found immunopositivity in nerve fibers in the wall of the gastrointestinal tract of all mammalian species examined, namely mouse, rat, hamster, guinea pig, ferret, cat, pig, sheep and man. In the gastric mucosa, they observed delicate PACAP-immunoreactive fibers in the mouse, rat, hamster and human, but not in the other species examined. Fine varicose fibers were found in the mucosa (both oxynthic and pyloric parts) and also in the muscularis layer in the rat stomach. Moderate number of PACAP-containing fibers was seen in the submucous ganglia, while numerous nerve fibers as well as immunopositive nerve cell bodies in the myenteric ganglia (11). PACAP is frequently colocalized with the sensory neuropeptide CGRP and also with VIP. In pigs, PACAP-immunoreactivity was described in beaded nerve fibers in all layers of the antrum, with higher number in the muscular and submucosal layers than in the mucosa. Furthermore, immunoreactivity was observed in nerve cell bodies of the myenteric ganglia, but not in the submucosal plexus (13). Colocalization with VIP was observed both in the fibers and in the ganglionic cells and some fibers costored CGRP, especially those innervating submucosal blood vessels (13). In the mucosa, only few PACAP positive nerve fibers could be found, mainly around blood vessels and some associated with basal glandular cells (13).

Other studies have also confirmed these findings. Kantor et al. (15) could detect PACAP-specific mRNA in the oxynthic mucosa of the rat stomach with RT-PCR. PACAP mRNA was also demonstrated by *in situ* hybridization in a few nerve cell bodies in the myenteric ganglia indicating some intrinsic synthesis of the peptide (11). Studying further the origin of PACAP-ergic nerves Hannibal et al. performed capsaicin-induced denervation as well as surgical denervation. They found that neonatal capsaicin treatment reduced the concentration of PACAP in the stomach by about one-third. This was mainly confined to the oxynthic part of the submucosa, where a reduced number of immunopositive nerve fibers was observed, while fibers remained unaffected in the mucosa, muscularis and myenteric ganglia. After surgical extrinsic denervation, a modest decrease was observed. These data proved that the origin of PACAP in the stomach wall is dual: both intrinsic and extrinsic. The extrinsic innervation is most probably sensory, also supported by the observations of PACAP in the jugular-nodose ganglion of the vagus nerve and dorsal root ganglia (16–18).

PACAP immunoreactivity was also studied in the sheep digestive tract (19). Fibers were mainly detected in the muscular layer of the stomach, including cardia, corpus, antrum, and pylorus, with pyloric sphincter showing very strong PACAP-ergic innervation (19). Scarse immunolabeled fibers were detected in the mucosa, mainly in the lamina muscularis mucosae. Fibers and few perikarya were detected in myenteric ganglia (19). Presence of PACAP in the stomach wall has also been confirmed in cats (20). In the guinea pig, myenteric fibers showed weak immunoreactivity, together with lamina propria around glands and submucosal blood vessels, with weaker expression than in other mammalian species (21). PACAP immunoreactivity showed similar pattern in another rodent, Mastomys stomach, where PACAP was found in the oxynthic mucosa between the glands and in the submucosa (22).

Based on the very limited available data, PACAP occurs also in the human stomach, similarly to the distribution in other mammals. As mentioned earlier, Sundler et al. (14) observed delicate PACAP1–27-like immunoreactive fibers in the stomach. Vincze et al. have described first the presence of PACAP in the normal human stomach (20). In addition to the few PACAP-positive fibers in the mucosa, numerous cells contain PACAP in the glands of the fundus and corpus, and less in the cardia and pylorus. Electron microscopical observations showed that mainly the parietal cells contained perinuclear PACAP immunoreactivity (20). During fetal development, PACAP immunoreactivity appears in the human gastric glands, of both corpus and pylorus, around the 18th to 20th intrauterine weeks (23).

The presence of PACAP has also been investigated in the alimentary tract, including the stomach, of several non-mammalian species. For example, PACAP/GHRH-like mRNA could be detected in the stomach of catfish (24). PACAP and receptor transcripts have been found in another fish [tilapia (25)]. In zebrafish, immunohistochemistry established the presence of gut neurons expressing PACAP in the proximal part of the developing gut from the first stage investigated (2 days postfertilization) and before regular motility was observed. At 5 days postfertilization, PACAP reduced the regular propagating wave frequency of gut motility. This suggests that both excitatory and inhibitory pathways develop at an early stage in the gut, independent of exogenous feeding. This supports physiological results that gut motility is under neuronal control during the period when regular motility patterns develop (26). PACAP mRNA has also been identified in the olive flounder pylorus (27). Among reptiles, Valiante et al. (28) found mRNA for PACAP in gastric glands with *in situ* hybridization and PACAP peptide with immunohistochemistry as well as immunoreactivity with antisera against all three PACAP receptors in the lizard stomach. In frog species, Olsson showed the presence of PACAP in the entire gastrointestinal tract, including the stomach, of the African clawed frog (29). He showed immunopositivity in all layers of the stomach, in the endocrine cells of the mucosa, and in nerve fiber bundles and ganglionic cells of the myenteric plexus. PACAP and VIP colocalization was observed in most places.

In birds, several studies have shown the presence of PACAP in the proventriculus (glandular part of avian stomach) and gizzard. Sundler et al. (14) studied PACAP1–27-like immunoreactivity in the digestive tract of chicken in addition to several mammalian species (see above). In the proventriculus, numerous PACAP-immunoreactive endocrine cells could be observed which were identical to the serotonin-containing cells storing gastrin-releasing peptide (14). Simon et al. (30) described PACAP gene expression increase in the glandular stomach in case of food restriction. In addition, nerve elements in other layers also contained immunoreactivity. Studying the ontogeny of PACAP-containing elements, Salvi et al. (31) found the first PACAP-immunoreactive elements at embryonic days 4.5–5 in the mesenchymal bud of the proventriculus/gizzard. After the pharyngeal appearance at E4, PACAP elements formed a weak network in the marginal mesenchymal zone of the stomach bud, followed by gradual appearance in myenteric and submucous plexuses (31). PACAP immunoreactivity has also been studied in another avian species by Mirabella et al. (32–34). The presence of both PACAP1–38 and PACAP1–27 was demonstrated, the former being the predominant form, in the gastrointestinal tract of the duck (32). They found PACAP immunoreactivity in neurons and fibers of the ENS, in endocrine cells and in the gut-associated lymphoid tissue, suggesting multiple roles of the peptide in the duck gastrointestinal system. The majority of mucosal ganglion cells in the proventriculus were shown to contain PACAP (33). In pigeons, the coexistence of PACAP/VIP was revealed in the stomach in NADPH-positive myenteric neurons, implying that the nitrergic nerve population of the pigeon gastrointestinal tract takes part in regulation of muscle motility as an inhibitory descending nerve pathway (34).

Even invertebrates contain PACAP-like immunoreactivity in their alimentary canal, including the areas corresponding to mammalian foregut/stomach regions. PACAP1–27 has been shown to be the dominant form of the peptide according to a RIA study, but immunoreactivity to both peptides could be shown in the foregut/gizzard regions of the earthworm *Lumbricus polyphemus* (35). PACAP-immunoreactive elements have been identified mainly in the ganglia supplying the alimentary canal in three annelid species: *Lumbricus terrestris, Eisenia fetida*, and *Lumbricus polyphemus* (36, 37). This has also been shown during earthworm development, along with the appearance of PAC1 receptor-like immunoreactivity in the subesophageal and other ventral cord ganglia (38, 39). Interestingly, and in concert with the well-known regeneration-promoting effect of PACAP (40, 41), significant increases in the concentration of PACAP-like compounds were found in the body wall, alimentary canal, and in coelomocytes during regeneration. The most characteristic morphological feature was the accumulation of immunolabeled neoblasts in the injured tissues, especially in the ventral nerve cord ganglion that initiates and mediates regeneration processes, including that of the digestive tract (42). Although no functional data are available, taken together, these morphological observations indicate that PACAP (or a PACAP-like peptide) occurs early during phylogeny, and is present not only in vertebrates, but also in the invertebrate alimentary tract.

Early receptor binding studies already detected PACAP binding in the stomach (43). Receptors for both PACAP and VIP have been identified in the stomach wall. In porcine antrum, mRNA for PAC1 and VPAC1 and 2 was identified (13), PAC1 and VPAC2 were mainly expressed in the muscular, but not in the submucosal/mucosal layers, while VPAC1 was found in all layers (13). In the gastric smooth muscle of guinea pigs VPAC2 receptor was found (44). Other studies have also shown PACAP receptors in different layers of the stomach. For example, VPAC2 receptors have been detected in the smooth muscle cells isolated from rabbit and guinea pig stomach (45), and others also detected VPAC2 receptors in the muscularis mucosae and the main muscularis layers of the mouse stomach, while no VPAC2 was found in other layers (46). PACAP binding has been shown in various pharmacological studies investigating motility (47–49). Enterochromaffin-like (ECL) cells express PAC1 receptor, and it is believed that PACAP from the myenteric neurons acts on these cells (50–52). It is well supported by some studies that ECL cells express PAC1 receptor, but not the VPAC receptors in rats and mice (53, 54), although all three receptors were found by others in Mastomys (22). All splice variants of the PAC1 receptors have been described on ECL cells (55).

**Figure 1:**
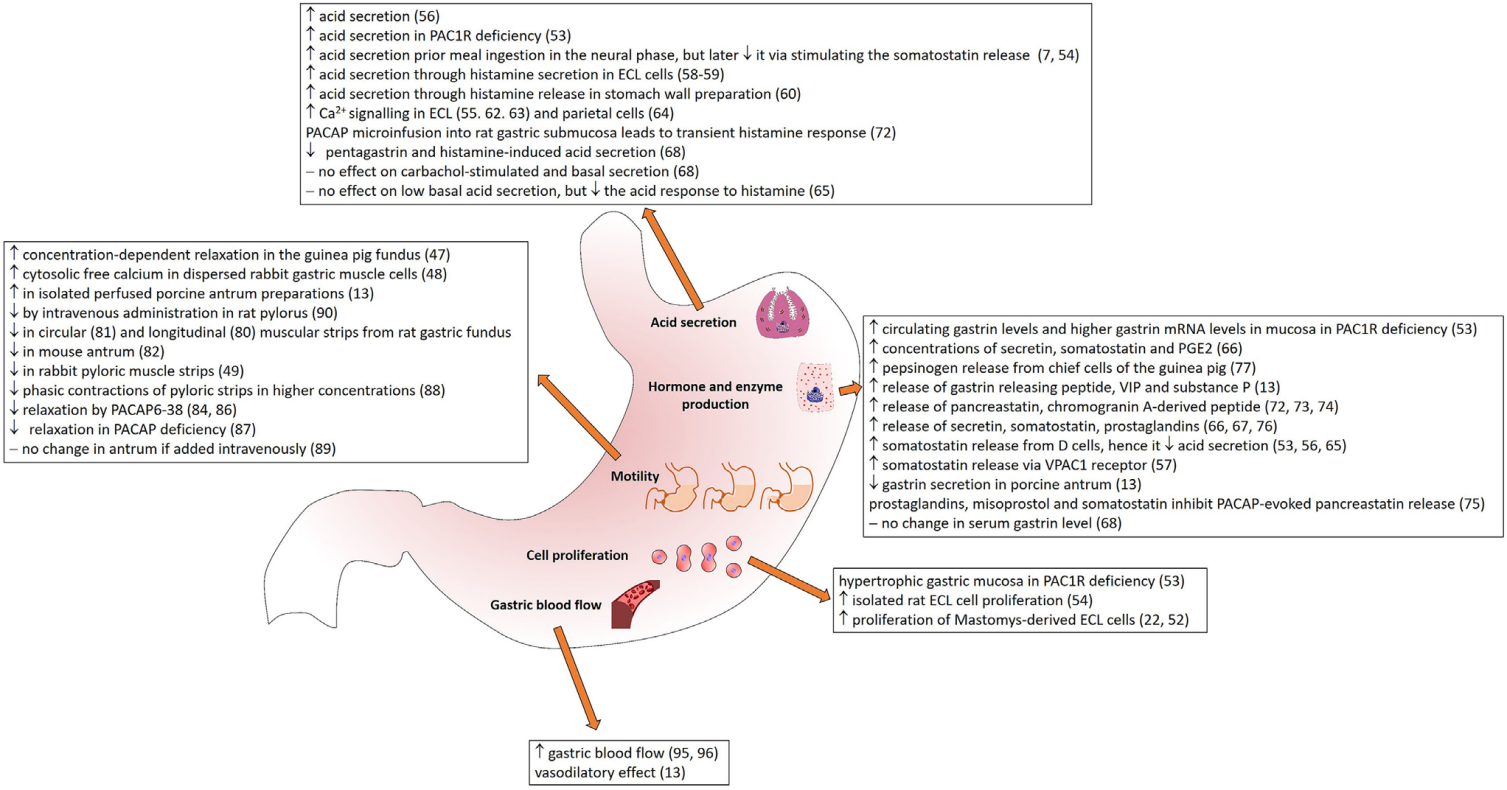
Summary of the effects of PACAP in stomach.

The wide distribution of PACAP and its receptors in secretory cells, in the nerves innervating different gastric layers and around blood vessels supplying the stomach (summarized in Table 1) suggests that PACAP plays various roles in gastric secretion, motility, and blood flow. In the following sections, we summarize currently available data on the different effects of PACAP in the stomach (Figure 1).

**Table 1:** Presence and main distribution sites of PACAP and its receptors in the stomach of various species.

Species	Localization of PACAP	Localization of PACAP receptor(s)	Reference
**VERTEBRATE**	**Dominant form: 1–38**		
**Mammalian**			

Cat	Nerve fibers of myenteric ganglia and muscularis		(14, 20)

Ferret	Nerve fibers in gastrointestinal wall		(14)

Guinea pig	Myenteric fibers, lamina propria, submucosal blood vessels, nerve fibers of muscularis	Gastric smooth muscle: VPAC2	(14, 21, 44, 45)

Hamster	Nerve fibers in mucosa, myenteric ganglia and muscularis		(14)

Human	Gastric glands, mainly parietal cells, nerve fibers of mucosa, myenteric ganglia, and muscularis		(14, 20, 23)

Mastomys	Oxynthic mucosa and submucosa	PAC1, VPAC1 and 2 on enterochromaffin-like cells	(22)

Mouse	Nerve fibers of mucosa, myenteric ganglia, and muscularis	Muscularis mucosae, muscularis: VPAC2 PAC1 on enterochromaffin-like cells	(14, 46, 53)

Pig	Nerve fibers in all layers of antrum, nerve cell bodies of myenteric ganglia, muscularis	Muscularis of antrum: PAC1, VPAC2 All layers of antrum: VPAC1	(13, 14)

Rabbit		Gastric smooth muscle: VPAC2	(45)

Rat	Submucous and myenteric ganglia, mucosa, submucosa, muscularis	PAC1 on enterochromaffin-like cells	(11, 14, 15, 50–52, 54)

Sheep	Muscularis of cardia, corpus, antrum and pylorus, muscularis mucosae, fibers, and perikarya of myenteric ganglia		(14, 19)

**Non-mammalian**			

Catfish	Stomach wall		(24)

Chicken	Endocrine cells in proventriculus, proventriculus, mesenchymal bud of proventriculus/gizzard, myenteric, and submucous plexus	PAC1 in proventriculus	(14, 30, 31)

Duck	Neurons and fibers of enteric nervous system, mucosal ganglionic cells in proventriculus		(32, 33)

Frog	All layers of gastric wall, endocrine cells of mucosa, myenteric plexus		(29)

Lizard	Gastric glands	PAC1, VPAC1, VPAC2	(28)

Olive flounder	Pylorus		(27)

Pigeon	Myenteric neurons		(34)

Tilapia		PAC1	(25)

Zebrafish	Gut neurons in proximal part of developing gut		(26)

**INVERTEBRATE**	**Dominant form: 1–27**		

Lumbricus polyphemus	Foregut, ganglia of alimentary canal		(35–37)

Lumbricus terrestris Eisenia fetida	Ganglia of alimentary canal		(36, 37)

## Effects of PACAP in the Stomach

### Effects on Gastric Secretion

As one of its main actions, PACAP leads to increases in cAMP levels also in the stomach (56). Interestingly, this action could not be shown in isolated stomach wall preparation under unchallenged conditions but was markedly potentiated in the presence of phosphodiesterase inhibitors, and it was four times greater than in the presence of the inhibitor alone, without PACAP addition (56).

A lot of work has been done in order to clarify the role of PACAP on acid secretion (Figure 1). In the beginning, several contradictory studies were published, some reporting stimulatory effects on acid secretion, while others inhibitory. The primary site for acid secretion is the parietal cells situated in the gastric corpus. In the cephalic phase, vagal afferents stimulate acid secretion through the release of acetylcholine and PACAP. The gastric phase of acid secretion is regulated by gastrin released from G cells, by histamine from ECL cells and somatostatin from D cells (7). The ECL cells control parietal cells by releasing histamine in their immediate vicinity. According to a number of studies, gastrin and PACAP stimulate histamine secretion from isolated ECL cells, while somatostatin and galanin inhibit stimulated secretion (57).

Plenty of studies show stimulatory actions of PACAP on acid secretion. PACAP increases acid secretion directly and also through stimulating histamine release (56). The histamine-releasing effect of PACAP in ECL cells has been shown *in vitro* (55, 58) and in stomach wall preparations (59). It has also been demonstrated that the PACAP-evoked secretion of histamine depends on Ca^2+^ entry (60). Several other studies have proven that through its receptor, PACAP activates calcium signaling and histamine release from ECL cells (55, 61, 62). Not only ECL cells respond to PACAP with elevated calcium levels, but also adjacent parietals cells (63). The PACAP-induced increase in gastric acid secretion can be blocked by the phosphodiesterase 4 inhibitor rolipram, which inhibits the degradation of cAMP (56).

The action of PACAP on gastric acid secretion has some controversial aspects. Although many studies describe that PACAP stimulates gastric acid secretion, the peptide is also known to stimulate somatostatin release from D cells (53, 56, 64), thus, it decreases gastric acid secretion. Li et al. described that the inhibition on gastric acid secretion in rat stomach by PACAP is mediated by secretin, somatostatin, and prostaglandin E2 (PGE2) (65, 66). Similarly, Mungan et al. found that intravenous PACAP inhibits pentagastrin and histamine-induced acid secretion in conscious pylorus-ligated rats and in gastric fistula rats, while no effect on carbachol-stimulated or basal secretion in pylorus ligated rats (67). Others have confirmed this observation: in wild-type urethane-anesthetized mice, PACAP1–38 did not affect the low basal acid secretion, but inhibited the acid response to pentagastrin, histamine, and bethanechol (64). Also, the involvement of somatostatin has been confirmed in this action, as in conscious wild-type mice, but not in somatostatin receptor 2 knockout mice, PACAP1–38 inhibited gastric acid secretion induced by 2-h pylorus ligation (64).

The contradictions have been partially resolved by studies showing that ECL cells possess PAC1 receptors, through which PACAP increases acid secretion, but also stimulates somatostatin release from D cells *via* VPAC1 receptor inhibiting gastrin and acid secretion through a less robust pathway (7, 54, 68–70). Pisegna’s group hypothesized that in the neural phase, PACAP increases acid secretion prior to meal ingestion, but later it inhibits it *via* stimulating the gastrin-inhibitory somatostatin release (7, 54). This theory is supported by the observations in PAC1-deficient mice: knockout mice develop gastric hypersecretion accompanied by elevated circulating gastrin levels and higher gastrin mRNA levels in the mucosa (53) in contrast to the findings of Mungan et al. (67), who detected no elevation in serum gastrin levels after intravenous PACAP administration. PAC1 receptor-deficient mice have elevated basal gastric output (nearly threefold increase compared to wild-type mice), have higher threshold to the effects of exogenous gastrin, but have an intact histamine stimulatory pathway (53). Another study has shown that microinfusion of PACAP into rat gastric submucosa produces a transient histamine response, in contrast to gastrin that shows a sustained action (71). The authors suggest that this transient response reflects receptor desensitization and/or depletion of secretory products (72). These observations can have translational value, as for example, PAC1 receptor knockout mice have been suggested to be a model for the human Zollinger-Ellison syndrome (65, 68). Also, the PACAP-driven regulatory mechanisms could play a role in several other clinical conditions related to disturbed gastric acid secretion (68).

As already mentioned earlier, PACAP affects release of several other factors. Stimulatory effects on somatostatin have been shown in porcine antrum where PACAP decreases gastrin secretion (13). ECL cells also produce pancreastatin in addition to histamine (71, 72). PACAP has been shown to evoke release of pancreastatin, a chromogranin A-derived peptide, with actions like inhibition of insulin secretion, pancreatic enzyme release, and gastric acid secretion (73). The PACAP-evoked release of pancreastatin can be inhibited by prostaglandins (72), galanin, misoprostol, and somatostatin (74). The stimulatory effects of PACAP on other substances have also been revealed. For example, PACAP stimulates the release of gastrin releasing peptide, VIP, and substance P (13). The release of somatostation, GRP, VIP and SP was not inhibited by PACAP6–38, suggesting the involvement of VPAC1 receptors, on which PACAP6–38 has no effect (13). PACAP also stimulates secretin release along with somatostatin and prostaglandins (65, 66, 75). PACAP is able to induce pepsinogen release from chief cells of the guinea pig (76). In isolated chief cells, PACAP and VIP binding sites were identified and PACAP1–38 induced biphasic pepsinogen release with the same potency as PACAP1–27 and VIP did. Li et al. (65) examined the inhibitory effect of PACAP1–27 on gastric acid secretion and its mechanism. It dose-dependently hindered both basal and pentagastrin-induced acid secretion. This inhibitory effect could be reversed using antisecretin, antisomatostatin serum, and indomethacin indicating that PACAP’s effect involves local release of secretin, somatostatin, and PGE_2_.

### Effects on Cell Proliferation and Differentiation

In isolated rat gastric ECL cells, Oh et al. (54) demonstrated a dose-dependent stimulation of proliferation, with 100 nM PACAP causing a maximum, 30% increase. Another study has also observed that PACAP stimulates proliferation of Mastomys-derived ECL cells, with 100 times more potency than VIP (22, 52). This could be inhibited by PACAP antagonist. In rabbit smooth muscle cells isolated from the antrum, no effect has been shown on the cell number in cultures, in contrast to colonic muscle cells, where PACAP inhibited the serum induced increase in cell number (77). This shows region-specific effects of PACAP on cell proliferation in the gastrointestinal tract. In PAC1 receptor deficient mice, hypertrophic gastric mucosa was observed with greater mucosal thickness resulting from greater gland height, but no difference in the pit sizes (53). This was accompanied by an increased cell mass, especially with increased parietal cell number, while total neuroendocrine cell number and D cell number was unaltered (53) (Figure 1).

### Effects on Motility

PACAP is known to have actions on smooth muscle of inner organs and blood vessels (78). In the stomach fundus, effects on motility resemble those in the esophagus (10, 47, 48). PACAP exerts similar, relaxant activity in the body and fundus of the stomach. In rat longitudinal gastric fundus muscle strips, a relaxant effect was produced by VIP, PACAP1–27, and secretin (79). Similarly, PACAP exerts relaxant activity on circular muscle strips from rat gastric fundus (80) and in the mouse antrum (81). In guinea pig gastric fundus, VIP, PACAP1-27 and PACAP1-38 induced concentration-dependent relaxation that was partly inhibited by the antagonists VIP10–28 and PACAP6–38 and the NO synthase inhibitor NG-nitro-l-arginine (l-NNA). Only relaxation induced by PACAP1–27 and PACAP1–38 was partly inhibited by apamin (47). Furthermore, electrical-field stimulation induced PACAP release. The authors conclude that the inhibitory transmission in guinea pig gastric fundus represents the combined actions of VIP, PACAP and NO released from nerve terminals and NO generated in muscle cells, which possess VPAC receptors, but no PAC1 receptor (47). PACAP binding was shown in dispersed rabbit gastric muscle cells, where PACAP, like VIP, stimulated cytosolic-free calcium and the formation of l-[3H]-citrulline, NO-3/NO-2, cGMP, and cAMP and induced relaxation (48). According to this latter study, the action of PACAP is mediated *via* the common VPAC receptors (48). PACAP also inhibits relaxation in the porcine lower esophageal sphincter (82). A recent study has confirmed the relaxant activity of PACAP in a dose dependent manner in mouse gastric fundus, while PACAP6–38 suppresses gastric relaxation (83). Evidence has also been published for the involvement of VIP/PACAP receptors in the afferent pathway mediating surgery-induced fundic relaxation in the rat (84) (Figure 1).

The role of endogenous PACAP in the regulation of motility is supported by the inhibition of the sustained relaxation by a PACAP receptor antagonist, PACAP6–38 (85). Furthermore, muscle strips prepared from PACAP knockout mice showed decreased level of sustained relaxation, which was about one-half of that observed in wild-type mice (85). PACAP6–38 inhibited electrical field stimulation-induced sustained relaxation (33.5% of control) in PACAP knockout mice. These findings were subsequently confirmed by others showing that mice deficient in PACAP have decreased relaxation in the stomach (86).

Pyloric sphincter muscle function is of utmost importance in gastric emptying, and its regulation is very complex, including regulation through nitric oxide, ATP in concert with nonadrenergic and noncholinergic transmission (87). Effects of PACAP on pyloric motility are somewhat contradictory. PACAP has been shown to increase motility of isolated perfused porcine antrum preparations (13). This could be blocked by PACAP6–38, which had no effect when applied alone. The relaxation in other parts of the stomach is not contradictory to the constriction in the pyloric part, since sphincter muscles in the GI tract are often antagonistically innervated or regulated. However, contradictory findings have also been reported. In dogs, intravenous administration of PACAP did not cause a change in antral motility using chronically implanted antral force transducers (88). Similarly, Ishiguchi et al. (87) found that lower concentrations of VIP and PACAP (nM) had no effect on isolated pyloric strips, only higher concentrations inhibited phasic contractions. Parkman et al. (49) also reported that VIP, PACAP1–38 and PACAP1–27 inhibited pyloric muscle in rabbit pylorus muscle strips. They also found an inhibitory effect of PACAP6–27 on both PACAP and VIP-induced relaxation, suggesting that PACAP and VIP act on the same receptor (49). *In vivo*, intravenous administration of PACAP (0.3–3 nmol/kg) caused significant relaxations of the rat pylorus suggesting that PACAP acts as inhibitory neurotransmitter in the rat pylorus and regulates gastric emptying (89). These discrepancies may be due to differences in species or experimental paradigms (13).

## Effects of PACAP on Gastric Blood Flow

PACAP-immunoreactive fibers are often observed around and in the walls of blood vessels. The general vasodilatory action of PACAP is well established in several species and experimental models (78, 90–93). This was also described for the stomach wall (13). Especially rich PACAP-ergic innervation could be observed in the submucosa, but the few PACAP-ergic nerve fibers observed in the mucosa were associated mainly with blood vessels, indicating a potential role in the blood supply of the stomach (13). Indeed, it has been confirmed that both PACAP1–27 and PACAP1–38 increase gastric blood flow, shown in the left gastric artery of dogs (94, 95), where PACAP resulted to be a potent vasodilator, stronger than VIP, in the gastric arterial bed *in vivo*. These studies applied the peptides intravenously in conscious dogs, previously implanted with flowmeters. The authors conducted similar experiments with PACAP fragments. They showed that C-terminal deletions or changing single amino acids in the N-terminal did not cause a change in the vasodilator capacity, but substituting amino acids 4 and 5 significantly changed the biological activity. Responses of the left gastric artery to Ala4, Val5-PACAP1–27 and VIP were similar, demonstrating that positions 4 and 5 are the key N-terminal residues for PACAP1–27 (94). These data indicate a physiological role of the peptide in the regulation of gastric blood flow.

## Changes of PACAP Expression Under Physiological and Pathological Conditions

Increased PACAP1–38 release has been observed in the porcine antrum upon vagus nerve stimulation and capsaicin treatment (13). As neonatal capsaicin treatment decreased PACAP content in rat stomach, PACAP is probably released from sensory nerve terminals (11). These observations are consistent with other studies in visceral organs, for example, similar results were obtained in the trachea: both capsaicin and electrical-field stimulation increased PACAP-release (96). PACAP or receptor upregulation has been observed in a few other conditions in the stomach: for example, CCK knockout mice show upregulated PACAP receptor expression in the mucosa, possibly indicating a compensatory process (97).

## PACAP in the Dorsal Vagal Nucleus in Gastric Pathological Conditions

PACAP can be observed under normal conditions in approximately 30% of the neurons in the dorsal vagal nucleus projecting to the prepyloric region in pigs (98). According to several studies, PACAP is one of the main neuropeptides in this nucleus, as its levels are higher under normal conditions than those of other examined neuropeptides (98, 99). In acetylsalicylic acid-induced gastritis, the number of PACAP-expressing neurons in the dorsal vagal nucleus increases by almost 50% in addition to *de novo* appearance of numerous other peptides, including VIP and galanin. These data suggest that neuronal PACAP is included in the mediation of the neural response to stomach inflammation (99). Similar results were obtained after partial gastric resection, a model of traumatic neuronal injury of the stomach: the number of PACAP-expressing neurons increased by 45%, along with increases of other neuropeptides (98). The authors suggest that the upregulation of PACAP implies its trophic and protective role in these gastric pathological conditions (98). These actions seem to be generally present in the digestive tract, as increases in PACAP-expressing neurons in the intestinal ganglia were reported both after axotomy and inflammation in the descending colon, in contrast to VIP, the levels of which did not change in these models (100, 101).

## PACAP in Gastritis and Ulcer

As PAC1 receptor deficient mice develop higher gastric acid production accompanied by gastric mucosal hypertrophy, it is suggested that the PACAP/PAC1 system plays an important role in gastric acid secretion not only under normal, but also under diseased conditions (53). Indeed, an earlier study found altered PACAP tissue expression during ulcer healing in rats (102). In a model of experimental ulcer, induced by local injection of acetic acid, PACAP and VIP immunohistochemistry was performed during the healing process. Starting on day 1, a marked depletion of PACAP immunoreactivity in nerve fibers at the margin of the ulcer was observed, again observed on days 10 and 15 (102). This was in contrast to VIP immunoreactivity, which did not show any alterations during the ulcer healing process. Immunoreactivity was also studied in the smooth muscle underlying the ulcer, where an upregulation of PACAP and VIP could be observed from day 10, along with an upregulation of PACAP and VIP mRNA in the myenteric ganglia in the ulcer’s neighborhood (102). This shows that neuronal PACAP depletion was transient and fully reversible. The authors argue that the selective decrease of PACAP at the ulcer margin might be due to either excessive release or a decrease in synthesis. Duodenal ulcers are linked to gastric acid-induced lesions and PACAP has been shown to have protective effects also in a duodenal ulcer model (103). The peptide is a known stimulant of duodenal bicarbonate secretion (104–107) and thus, can protect against the irritant effects of gastric acid. Indeed, intravenous bolus injection or infusion of PACAP1–27 increased duodenal bicarbonate secretion even in the presence of mepirizole, without an effect on acid secretion, and significantly reduced the severity of duodenal lesions (103).

## PACAP and Stomach Cancer

PACAP has been shown to play a role in cell proliferation and differentiation in numerous normal and tumorous cells and expression of PACAP or its receptors is suggested to correlate with tumor growth and differentiation (108–110). It has also been shown that expression of the peptide and/or its receptors show marked changes in certain tumor types either in the tumor itself or in peritumoral areas (111–115). Little is known about the connection between gastric tumors and PACAP, but some data indicate that PACAP may play a regulatory role in gastric tumor biology. Studying human tumors, it has been revealed that PACAP receptors are expressed in gastric cancer. Overexpression of PACAP receptors has been reported in various types of cancer, including stomach cancer in about half of the examined cases (114–117). Schulz et al. could detect VPAC1 and VPAC2 receptors in gastric cancer (118, 119), while they could not detect PAC1 expression in gastric tumors.

PACAP is linked to proliferative signaling pathways and tumor growth (120). It was proposed that, similarly to solid tumors, PAC1 receptors are expressed on neuroendocrine tumor cells and may mediate biological effects induced by PACAP, such as secretion and growth (120). In Mastomys ECL cells, transformation was induced by histamine 2 receptor blockade (22). During the process of mucosal hyperplasia induced by endogenous hypergastrinemia, PACAP-LI increased significantly and was identified primarily in a linear-punctuated pattern. In the stage of carcinoid tumor formation, PACAP-LI was present in striking abundance and mostly presented in the basket-like pattern. Indeed, most of the basket-like IR was identified within neoplastic lesions. In the fundus, PACAP immunoreactivity significantly increased in the tumor mucosa compared to controls (22). Investigation of the receptor subtypes revealed that the expression levels of PAC1 and VPAC2 were modestly upregulated in tumor ECL cells compared with naive cells, but that of VPAC1 receptor subtype appeared to be downregulated (22). PACAP induced the proliferation of transformed ECL cells, and this effect was stronger than that of TGFα or gastrin (22). These effects could be completely antagonized by PACAP6–38 and to a lesser extent by a VPAC1 antagonist (22). Although it is not known whether this process plays a role in human gastric tumor formation, these results indicate that PACAP potently modulates ECL cell proliferation and is involved in ECL cell transformation. The growth of the neuroendocrine-derived ECL cells in the stomach has been shown to be influenced by PACAP (121). Lieu et al. (120) have demonstrated that PAC1 receptors are expressed in the well-established neuroendocrine cell-derive BON cell line. The authors propose that PACAP may regulate the biological release of peptides and serotonin from BON cells and that, like in solid tumors, PACAP could potentially stimulate the growth of BON cells (120). These few data indicate that PACAP may play a role in the growth of different types of stomach cancer.

## Motility Disorders

In spite of the large amount of data regarding actions of PACAP on motility, very little is known about motility disorders associated with PACAP signaling. The role of endogenous PACAP in gastric motility is supported by observations in PACAP KO mice: relaxation effect of PACAP is reduced by about 50% in muscle strips prepared from PACAP knockout mice (85). These findings were subsequently confirmed by others: mice deficient in PACAP have decreased relaxation in the stomach (86). Although no *in vivo* data are available for PACAP, VIP knockout mice have been shown to develop intestinal motility dysfunction, similar to that observed in human Hirschprung’s disease (122). Furthermore, dystrophic mice have been reported to develop gastrointestinal motor disorders, where PACAP6–38 abolished off-relaxations and also caused a reduction in amplitude of the contractile responses and efficacy of PACAP 6–38 on the excitatory responses was lower in strips from dystrophic mice than in wild types (123). The observed stronger peptidergic modulatory action can contribute to the altered gastric contractile responses in this motility disorder (123). Whether these alterations are also present in human conditions, awaits further investigations.

## Author Contributions

DR, AI, BO, ES, AT, and GH have collected data in the literature in order to give an accurate review.

## Conflict of Interest Statement

The authors declare that the research was conducted in the absence of any commercial or financial relationships that could be construed as a potential conflict of interest.
